# Attentional Bias to Angry Faces: Contrasting Responses in Typically Developing Children and Children with Autism Spectrum Disorder

**DOI:** 10.1192/j.eurpsy.2024.949

**Published:** 2024-08-27

**Authors:** S.-Y. Kim, Y. Choi

**Affiliations:** Psychology, Duksung Women’s University, Seoul, Korea, Republic Of

## Abstract

**Introduction:**

Human faces generally attract immediate attention. However, it has been found that children with autism spectrum disorder (ASD) tend to allocate relatively less attention to faces. Previous research showed that typically developing children (TD) exhibited an attentional bias to angry faces, regardless of their anxiety levels, but it’s unclear if this applies to children with ASD. Therefore, the present study aims to investigate attentional bias induced by angry and/or happy faces in children with ASD.

**Objectives:**

We explored attentional bias toward angry faces in both TD children and children with ASD. We hypothesize that while TD children will show attentional capture effects in response to angry faces, children with ASD will not exhibit such attentional bias to facial stimuli, irrespective of their emotional content.

**Methods:**

By now, five ASD participants (all male) and 34 TD participants (17 male), aged 6-12, have completed a continuous performance task. In this task, irrelevant distractors (angry or happy faces) appeared and disappeared abruptly, while the orientation of the target changed every 1,250 ms. Participants were asked to respond as quickly and accurately as possible to the orientaiton of the target. We designated the time when the distractor first appeared as T1, and subsequent time intervals at 1,250 ms increments were labeled as T2, T3, and T4. The time intervals when no distractor was present were labeled as TB (baseline). If the reaction time (RT) at T1 was significantly slower compared to TB, it indicated attentional bias by the distractor.

**Results:**

For the RT data, separate repeated measures ANOVAs with 2 (emotion) * 5 (time) factors were conducted for each group. The results revealed a significant main effect of time (*F*(4, 132) = 17.59, *p* < .01) and a significant interaction between emotion and time (*F*(3.27, 107.74) = 4.92, *p* < .01) only in TD. Post hoc t-tests indicated that TD children exhibited significantly slower RT at T1 compared to TB, but this difference was observed only for angry faces (*t*(33) = 4.84, *p* < .01). In contrast, no significant effect was found in children with ASD. In other words, TD demonstrated attentional bias only when exposed to angry faces, while ASD children did not exhibit attentional bias to either emotion.

**Conclusions:**

This study aimed to investigate attentional bias to angry faces in both TD and ASD children. The results indicate that TD children exhibited an attentional bias when exposed to angry faces, whereas ASD children did not display such bias. These findings are consistent with previous research suggesting that TD children tend to show attentional bias towards angry faces, regardless of their anxiety levels. Furthermore, the absence of attentional bias to angry faces in ASD suggests that their characteristic of reduced attention to faces may contribute to the lack of attentional bias towards angry faces.

**Disclosure of Interest:**

None Declared

